# A new model to simulate and analyze proliferating cell populations in BrdU labeling experiments

**DOI:** 10.1186/1752-0509-7-S1-S4

**Published:** 2013-08-12

**Authors:** Daniella Schittler, Frank Allgöwer, Rob J De Boer

**Affiliations:** 1 Institute for Systems Theory and Automatic Control, University of Stuttgart, Pfaffenwaldring 9, 70569 Stuttgart, Germany; 2 Department of Theoretical Biology, Utrecht University, Padualaan 8, 3584 CH Utrecht, The Netherlands

## Abstract

**Background:**

This paper presents a novel model for proliferating cell populations in labeling experiments. It is especially tailored to the technique of Bromodeoxyuridine (BrdU), which is taken up by dividing cells and thus accumulates with increasing division number during uplabeling. The study of the evolving label intensities of BrdU labeled cell populations is aimed at quantifying proliferation properties such as division and death rates.

**Results:**

In contrast to existing models, our model considers a labeling efficacy that follows a distribution, rather than a uniform value. It thereby allows to account for noise as well as possibly space-dependent heterogeneity in the effective label uptake of the individual cells in a population. Furthermore, it enables more informative comparison with experimental data: The population-level label distribution is provided as a model output, thereby increasing the information content compared to existing models that give the fraction of labeled cells or the mean label intensity.

We employ our model to study some naturally arising examples of heterogeneity in label uptake, which are not covered by existing models. With simulations of noisy and spacially heterogeneous label uptake, we demonstrate that our model contributes a more realistic quantitative description of labeling experiments.

**Conclusion:**

The presented model is to our knowledge the first one that predicts the full label distribution for BrdU labeling experiments. Thus, it can exploit more information, namely the full intensity distribution, from labeling measurements, and thereby opens up new quantitative insights into cell proliferation.

## Introduction

The proliferation of cells is a central process in biology and life, and as such a major topic in systems biology. Labeling techniques using, e.g., Bromodeoxyuridine (BrdU), deuterium, or Carboxyflourescein succinimidyl ester (CFSE), are successfully applied to study cell proliferation. Mathematical models have been developed especially for these labeling experiments and enable the quantitative evaluation of cell proliferation data [1-5]. BrdU is a DNA label that is commonly applied *in vivo *over a time span of days or weeks, during which it is taken up by proliferating cells. Thus it slowly accumulates in the cell with increasing number of divisions during this so-called "uplabeling". Spatial heterogeneity with respect to the label concentration throughout the organism, temporal variations due to label dosing over weeks, as well as the stochasticity inherent to biochemical processes, may cause varying label uptake in cells [1]. The significance of heterogeneity in label uptake is supported by experimental data [6], where cells of similar division number indeed show varying label intensities. This shows that there is no trivial relation that would allow to deduce proliferation properties directly from label intensities.

Existing models for BrdU-labeling [1,4,7] assume a uniform value of label uptake. Thus they fail to reproduce a realistic label distribution, since a uniform labeling value would result in sharp peaks each corresponding uniquely to a certain number of undergone divisions. What is more, they neither reflect noise in label uptake, nor spatial heterogeneity such as label concentrations differing between organs, nor temporal label variations.

This paper presents a novel, more general model for label distributions in proliferating cell populations, which overcomes these drawbacks and contains existing models as a special case. The mathematical model presented here is the first to the authors' knowledge to account for heterogeneity in label uptake. The model is formulated for general labeling efficacy distributions, such that it can reflect noise, time intervals with different labeling conditions, as well as heterogeneity arising, e.g., from spatially different label concentrations.

The general model is developed in the Methods section. We demonstrate how our model serves to simulate relevant examples of noisy and spatially heterogeneous labeling conditions in the Results section. A short summary and discussion is provided in the Conclusions.

**Notation: **In this paper, *p *(*x|y*) denotes the probability density of property *x*, given *y*, with the integral over the whole domain equal one. *n*(*x|y*) denotes the number density of property *x*, given *y*. *δ*(*x*) denotes the Dirac delta distribution. |A| denotes the cardinality of a set A . $e_{k}^{T}$ denotes the unit row vector with *k*-th entry one.

## Methods

In order to develop a model for the label distribution of proliferating cell populations, we choose an intuitive approach of modeling two separate processes: The dynamics of proliferating cells are given by a population model describing cells by their division number (hence, called "division-structured"), whereas for the label content expected after a certain number of divisions we introduce a novel label distribution model. Finally, both are combined into a model for the cell population's label distribution.

### Modeling the number of cells

To model the number of cells that have divided a certain number of times, we make use of an established division-structured population model [3,5], and extend it to a finite number of subsequent time intervals. This is important since BrdU labeling experiments comprise at least two, but possibly more, time intervals with different labeling conditions (e.g., uplabeling and delabeling phase), as will be exemplified later. Let these time intervals be given by (*T*_*k*-1_, *T_k_*], with *T*_0 _= 0 and positive $T_{k}\in {\mathbb{R}}_{++}$, and with increasing time points *T*_*k*-1 _<*T_k_*, for all *k *= 1, : : : , *K*. The vector **i **= [*i*_1_, . . . , *i_K_*] denotes a sequence of division numbers during these time intervals. Then, the model's state variable *N*(**i|***t*) represents the number of cells which have divided *i_k _*times during the time intervals (*T*_*k*-1_, *T_k_*], respectively, at a certain time *t*. The dynamics of *N*(**i**|*t*) are given by a system of ordinary differential equations (ODEs), $\forall \text{i}\in {\mathbb{N}}_{0}^{K}$:

(1) $$\begin{array}{c} \forall t\in \left(T_{k-1},\;T_{k}\right]:\\ \begin{array}{ccc} \frac{dN\left(i|t\right)}{dt} & = & -\left(\alpha_{i}\left(t\right)+\beta_{i}\left(t\right)\right)N\left(i|t\right)\\ & + & \left\{\begin{array}{cc} 0 & ,\;i_{k}=0\\ 2\alpha_{i-e_{k}^{T}}\left(t\right)N\left(i-e_{k}^{T}|t\right) & ,\;i_{k}\geq 1 \end{array}\right) \end{array} \end{array}$$

where *α***_i_**(*t*) denotes the cell division rate, *β***_i_**(*t*) the cell death rate, at time *t *of cells which have divided **i **times, and with initial conditions

(2) $$N\left(i|0\right)=\left\{\begin{array}{ccc} N_{\text{ini}} & , & i=0^{T}\\ 0 & , & i\neq 0^{T} \end{array}\right)$$

which means that at the beginning of the experiment, no cells have divided yet in the presence of label. For several cases there exist analytical solutions for this ODE system, which have been reported elsewhere [3,5]. Moreover, several extensions for this model are available, for example if cell types [8] or age structure [9] are of importance, which allows to generalize the model class to cover a wide range of biological systems. Instead of the infinite dimensional ODE system (1), it is often desirable to use a finite dimensional ODE system similar to (1) but with **i ∈**{0, . . . , *S*}*^K^*. This allows for example an efficient numerical solution of the ODE system. The accompanying truncation error can be made arbitrarily small by choosing a sufficiently high *S *[5]. With this broadly general model class at hand, one can pick an appropriate model when analyzing a specific biological cell population system.

### Modeling the label content

Next, we develop a model for the label content of cells in dependence of undergone divisions. Then, in combination with the model of cell numbers we derive a model for the emerging label distribution in the overall cell population.

For BrdU labeling, at least two time intervals need to be distinguished: During uplabeling, the label is introduced into the organism and therefore incorporated at each cell division into the newly synthesized DNA strands. In contrast, when the label is withdrawn from the organism the label content of a cell is diminished upon each division.

#### Labeling efficacy

Let the random variable $U_{i}^{\left(k\right)}$ denote the amount of incorporated label for newly built DNA strands, *i *received at a particular *i*-th cell division event that takes place in the time interval (*T*_*k*-1_, *T_k_*]. This random variable is drawn from a labeling efficacy distribution: $U_{i}^{\left(k\right)}\sim p_{\text{eff}}^{\left(k\right)}\left(x\right)\in {\mathbb{R}}_{+}$, which is a probability density, defined over possible values of label content x∈ℝ. This probability density will determine the label content of newly built DNA, as we will see in the following.

#### Label content of an individual cell

The label uptake during subsequent divisions then resembles a repeated sampling from this distribution for the newly built DNA strands. These newly labeled strands make up half of the total DNA of each daughter cell, such that the cell receives $\frac{1}{2}U^{\left(k\right)}\sim 2p_{\text{eff}}^{\left(k\right)}\left(2x\right)$ label content. The old labeled strands can be viewed as about equally distributed to both daughter cells: Since cells contain typically 46 (humans) or 42 (rhesus macaques) chromosomes, it is unlikely that two daughter cells receive strongly deviating amounts of labeled strands from the same mother cell. If we denote the current label content of a dividing cell by *X*(*i*), its two daughter cells receive via the old strands the label content $\frac{1}{2}X\left(i\right)$. Taken together, for one time interval, the label content of a new cell is assembled from label contents of old and new strands as $X\left(i+1\right)=\frac{1}{2}U_{i+1}+\frac{1}{2}X\left(i\right)$, where *X*(*i*) denotes the label content of a cell after *i *divisions. With this recursive definition and *X*(0) = 0, we arrive at $X\left(i\right)=\sum_{l=1}^{i}2^{-j}U_{j}$ for the label content after *i *divisions. Note: The special case of $p_{\text{eff}}^{\left(k\right)}\left(x\right)=\delta \left(x-\mu \right)$ represents a uniform labeling value as used in previous models [4].

Label dilution during delabeling (i.e., when there is no label in the environment) can be modeled as the special case of zero labeling efficacy, *p *_eff _(*x*) = *δ*(*x*). Then, the label content of cells during delabeling gets halved upon each division. In summary, considering *K *time intervals, the label content after **i **divisions during the time intervals (*T*_*k*-1_, *T_k_*] with *k *= 1, . . . , *K *is given by the random variable $X\left(i\right)=\sum_{k=1}^{K}\left(\sum_{l=1}^{i_{k}}2^{-\left(l+\Sigma_{r=j+1}^{K}\;i_{r}\right)}U_{l}^{\left(k\right)}\right)$. For example, for two time intervals, the label content after *i*_1 _divisions during uplabeling and *i*_2 _divisions during delabeling is given by $X\left(i_{1},\;i_{2}\right)=2^{-i_{2}}\sum_{j=1}^{i_{1}}2^{-i_{1}}U_{j}^{\left(1\right)}$. In general, the random variable of label content follows a distribution *X*(**i**) ~ *p *(*x|***i**). This distribution can be obtained from the labeling efficacy distributions, $p_{\text{eff}}^{\left(k\right)}\left(x\right)$, recursively as follows, for all $i\in \mathbb{N}\times {\mathbb{N}}_{0}^{K-1}$:

(3) $$\begin{array}{c} X\left(i\right)\;\sim \;p\left(x|i\right)\\ \;\;=2\int_{-\infty }^{x}2p_{\text{eff}}^{\left(k\right)}\left(2x-2\chi \right)p\left(2\chi |i-e_{k}^{T}\right)d\chi , \end{array}$$

and with *p *(*x|***0^T^**) = *δ*(*x*). That is, $p\left(x|i\right)\in {\mathbb{R}}_{+}$ is a probability density defined over values x∈ℝ.

### Overall label distribution of the population

The output of labeling experiments consists of a sample of measured label intensities {*l*^(*v*)^}, *v *= 1, . . . , *M*, with *M *the number of measured cells. This corresponds to a sample from the overall label distribution. Typically this distribution sample is collapsed into the fraction of labeled cells, |{*l*^(*v*)^*|l*^(*v*) ^≥ *l_θ_*}|/*M *, by setting a threshold *l*_θ _above the background signal. Some additional information can be exploited by computing the mean fluorescence intensity, $\sum_{v=1}^{m}l^{\left(v\right)}/M\;\left[1,4\right]$.

The fraction of labeled cells or the mean fluorescence intensity is the maximum information that is used by existing models to infer proliferation parameters from BrdU data [1,4,7]. This means that the detailed information, how many cells contain how much label, is available in the data but not exploited in these models. What is more, this thresholding approach has the severe drawback that the fraction of labeled cells is highly sensitive to the value of the threshold, which can usually not be determined exactly, impeding a reliable estimation of proliferation parameters.

A considerable advantage of our model is that the complete label distribution is indeed available as a model output. This provides a more detailed and less sensitive option for fitting a model to the data and inferring proliferation parameters. We will shortly outline how the overall label distribution *m*(*x|t*) is obtained in our model.

The probability of observing at time *t *a cell with label intensity *x *and division number sequence **i **is expressed by the number density *n*(*x*, **i***|t*), which is defined over$\left(x,\;i\right)\in \mathbb{R}\times {\mathbb{N}}^{K}$. These number densities in turn can be assembled as

(4) n(x,i|t)=N(i|t)⋅p(x|i)

where *N*(**i|***t*) are the solutions of the ODE system (1), and *p *(*x|***i**) are the probability densities (3). The dynamics of *n*(*x*, **i|***t*) can be written as a system of partial differential equations (PDEs):

(5) $$\begin{array}{c} \begin{array}{ccc} \frac{\partial n\left(x,i|t\right)}{\partial t} & = & -\left(\alpha_{i}\left(t\right)+\beta_{i}\left(t\right)\right)n\left(x,\;i|t\right) \end{array}\\ +\left\{\begin{array}{cc} 0 & ,\;i_{k}=0\\ 4\alpha_{i-e_{k}^{T}}\left(t\right)\int_{-\infty }^{x}2p_{\text{eff}}^{\left(k\right)}\left(2x-2\chi \right). & \\ \;\;\;\;\;n\left(2\chi ,\;i-e_{k}^{T}|t\right)d\chi & ,\;i_{k}\geq 1 \end{array}\right) \end{array}$$

which solution *n*(*x*, **i***|t*) can be shown to be equivalent to (4).

Finally, these individual number densities can be summed up over all division numbers to arrive at the overall label distribution:

(6) $$\begin{array}{cc} m\left(x|t\right) & =\underset{i\in {\mathbb{N}}_{0}^{K}}{\sum }n\left(x,i|t\right)=\overset{K}{\underset{k=1}{\sum }}\overset{\infty }{\underset{i_{k}=0}{\sum }}n\left(x,\;i|t\right)\\ & =\overset{K}{\underset{k=1}{\sum }}\overset{\infty }{\underset{i_{k}=0}{\sum }}N\left(i|t\right)p\left(x|i\right). \end{array}$$

Using the truncated model, this becomes a finite sum

(7) $$\tilde{m}\left(x|t\right)=\overset{K}{\underset{k=1}{\sum }}\overset{S}{\underset{i_{k}=0}{\sum }}N\left(i|t\right)p\left(x|i\right)$$

which can be made arbitrarily close to the infinite sum *m*(*x|t*) by sufficiently high *S*, which are usually in the moderate order of *S *≈ 20.

Achieving the label distribution via the decomposed approach (4) has the clear advantage that, instead of solving a PDE system, one only has to solve a system of ODEs, and convolutions of probability densities. Circumventing the need to solve a system of PDEs offers a more efficient solution and simulation.

## Results

We now demonstrate the value of the presented model by studying two relevant scenarios of labeling, which are not covered by any existing models that we are aware of. The assumed cell population properties are the same for both scenarios, which only differ in the labeling conditions. We simulate a hypothetical cell population of 1000 cells with division and death rates *α_i_*(*t*) = *α *= *β_i_*(*t*) = *β *= 0.1[1/*d*]. An uplabeling phase of 10 days is assumed, with labeling efficacy specifications as pointed out in the respective scenario. The labeling conditions are now specified in the respective scenario, and the arising label distribution is investigated.

### Noise in label uptake

An important property of biochemical processes is their inherent noise and resulting uncertainties. This can also be expected to be present in label uptake, for example because the local concentrations of label molecules are exposed to stochastic fluctuations. These deviations from the expected label uptake can be reflected by a normally distributed labeling efficacy $N\left(u|1,\;\sigma^{2}\right)$. The mean *μ *= 1 denotes the expected label uptake, and the standard deviation *σ *determines the magnitude of noise in the label uptake. To illustrate a simple scenario with noisy label uptake, we assume two time intervals: Firstly the uplabeling phase with a normal distributed labeling efficacy, $p_{\text{eff}}^{\left(1\right)}\left(u\right)=N\left(u|1,\;\sigma^{2}\right)$, and secondly the delabeling phase with zero labeling efficacy $p_{\text{eff}}^{\left(2\right)}\left(u\right)=\delta \left(x\right)$. Due to the properties of convolutions of normal- and Dirac delta distributions, the arising probability densities can be analytically expressed as

(8) $$p\left(x|i_{1},\;i_{2}\right)=N\left(x|\frac{1-2^{-i_{1}}}{2^{i_{2}}}\mu ,\;\frac{\sum_{j=1}^{i_{1}}2^{-2j}}{2^{i_{2}}}\sigma^{2}\right).$$

**Figure 1:**
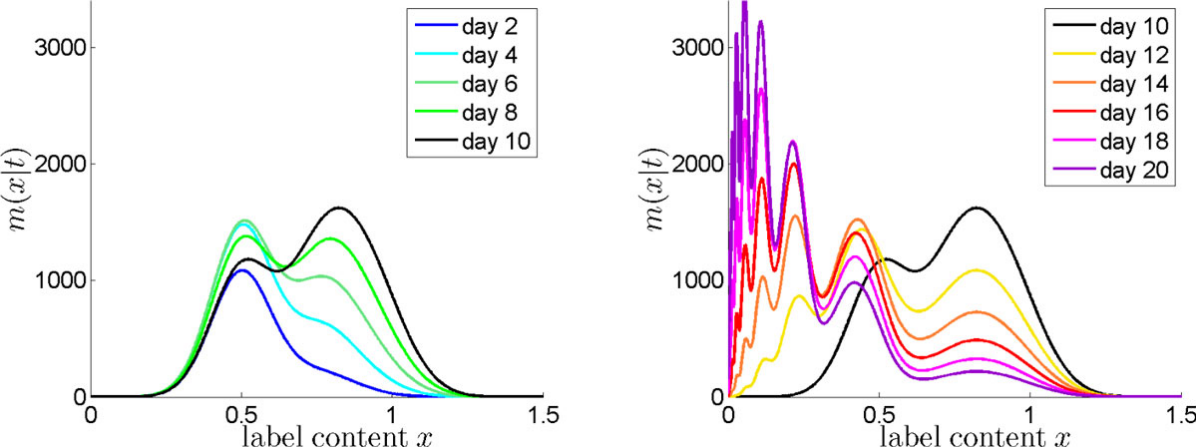
**Noisy label uptake**. Simulations of a proliferating cell population, assuming noise in labeling efficacy. Predicted overall label distribution *m*(*x|t*) during uplabeling (left), and during delabeling (right). A noise level of *σ *= 0.2 is assumed.

**Figure 2:**
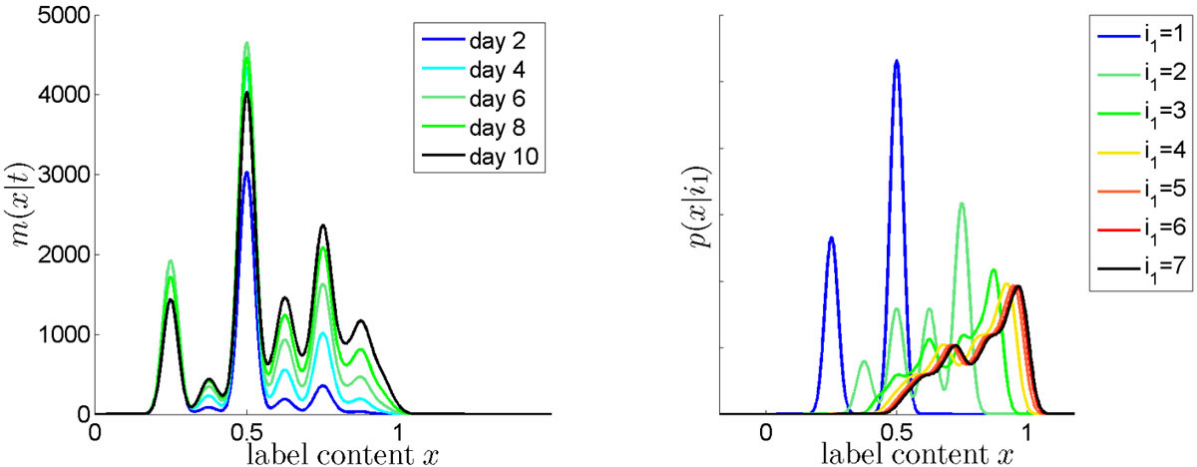
**Spatially heterogeneous labeling efficacy**. Simulations of a proliferating cell population, assuming two compartments with different labeling efficacy. Predicted overall label distribution *m*(*x|t*) during uplabeling (left), and probability densities *p *(*x|i*_1_) for particular division numbers *i*_1 _= 1 . . . 7 (right). For illustrative reasons, a low standard deviation within each compartment *σ *= 0.05 is assumed.

The obtained label distribution is shown in Figure 1 for *σ *= 0.2. The left panel in Figure 1 depicts the simulated overall label distribution in the cell population during uplabeling. Although the population and thus its label distribution is composed of cells with (more than two) different division numbers, the distribution exhibits only one peak (day 2, 4) or two peaks with a strong overlap (day 6, 8, 10). This highlights that under such labeling conditions, it is hardly possible to determine the number of cells that have divided once apart from cells that have divided more than once. The right panel in Figure 2 shows the overall label distribution during delabeling. In this phase, separated peaks occur, but only at very low label intensities. These may, in real experiments, well lay below the detection threshold and thus not appear in the data [4].

Overall, these simulations reveal that noise in label uptake is a potential reason for the fact that BrdU intensity profiles in experimental data do not show separable peaks, and thus can hardly be interpreted in a straightforward way. It illustrates that, in order to make the estimation of proliferation parameters robust against noise, it will be important to explicitly model the full intensity distribution.

### Spatially heterogeneous labeling efficacy

Besides noise in label uptake, our model can also handle more general distributions as they may arise, for example, from spatial heterogeneities. A possible scenario is that in certain organs or tissue the label concentration deviates. Then, cell division events result different label uptakes depending on the location where they take place.

We consider a simple scenario in which the organism is partitioned into two compartments with different labeling efficacy. Two thirds of all cell divisions are assumed to happen in locations with full labeling efficacy, whereas one third of cell divisions happens in locations with only half of the labeling efficacy:

(9) $$p_{\text{eff}}\left(x\right)=\frac{1}{3}N\left(x|0.5,\;\sigma^{2}\right)+\frac{2}{3}N\left(x|1,\;\sigma^{2}\right).$$

The simulation results are depicted in Figure 2. The left panel shows the overall label distribution *m*(*x|t*) in the cell population during the uplabeling phase. The distribution exhibits multiple peaks, which importantly do not correspond to different division numbers but instead result from cell division events in different compartments.

The right panel of Figure 2 depicts the individual probability densities *p *(*x|i*_1_) for different division numbers *i*_1_. According to the probability density, a cell that has divided can exhibit highly different values of label content, depending on whether and which of its divisions happened in the compartment with high or low labeling efficacy. In contrast, the probability density for a cell of division number 3 and that of division number 7 are almost identical. This means that cells of highly different division number can have similar label intensity, whereas two cells of the same division number can possess highly differing label intensities. In summary, this demonstrates that if labeling efficacy is spatially heterogeneous, from the label content of a cell one can not conclude about the number of undergone divisions. Again, this points to a possible reason why BrdU intensity data may impede the direct extraction of division numbers, and suggests that this may be partly overcome by considering whole BrdU distributions in modeling.

Although this example considers a simple compartmental setup, more complex heterogeneities can be easily realized by using a corresponding distribution for the labeling efficacy. Our model can thereby achieve the probability densities, given arbitrary distributions, by solving *S^K ^*convolution integrals (3).

## Conclusions

By modeling the label uptake as a random variable sampled from a distribution, rather than by a uniform value, our model can cover noise, heterogeneities, and temporal variations simultaneously. This creates several degrees of flexibility in our model, which enables a more realistic mathematical description of the biological labeling process. Importantly, it explicitly provides the population's label distribution as model output, which can be compared to experimental data in more detail. This renders our model less sensitive against uncertainties, and enables to exploit the full information from data. Yet, the model is computationally efficient, as the system of ODEs is easy to solve, and the probability densities can be obtained from convolutions which may be solved numerically or even analytically.

As we demonstrated in the Results section, simulations of labeled cell populations assuming noise or heterogeneity in label uptake predict label distributions that qualitatively resemble experimental data. This suggests that these factors potentially may play a crucial role for the readout of DNA labeling assays, and should be incorporated in models. Our model is thereby able to take noise, heterogeneity, and temporal variations in labeling into account.

While this paper focused on the detailed presentation of the new model, the authors plan to use the presented model in future work to estimate cell proliferation parameters from real data which was out of the scope of this contribution. This will eventually reveal whether noise in label uptake needs to be considered for the correct interpretation of BrdU labeling data. If so, the presented model promises a more reliable estimation of proliferation parameters and offers new quantitative insights about cell proliferation.

## Competing interests

The authors declare that they have no competing interests.

## Authors' contributions

DS and RDB designed the problem formulation, developed the mathematical methods, and implemented the numerical simulations. DS, FA and RDB wrote the article. All authors read and approved the final manuscript.

## Declarations

The publication costs for this article were funded by the German Research Foundation (DFG) within the funding program Open Access Publishing at the University of Stuttgart.

This article has been published as part of BMC Systems Biology Volume 7 Supplement 1, 2013: Selected articles from the 10th International Workshop on Computational Systems Biology (WCSB) 2013: Systems Biology. The full contents of the supplement are available online at http://www.biomedcentral.com/bmcsystbiol/supplements/7/S1.
